# How 10 at% Al Addition in the Ti-V-Zr-Nb High-Entropy Alloy Changes Hydrogen Sorption Properties

**DOI:** 10.3390/molecules26092470

**Published:** 2021-04-23

**Authors:** Jorge Montero, Gustav Ek, Laetitia Laversenne, Vivian Nassif, Martin Sahlberg, Claudia Zlotea

**Affiliations:** 1 University Paris Est Créteil, CNRS, ICMPE, UMR 7182, 2 rue Henri Dunant, 94320 Thiais, France; 2 Department of Chemistry–Ångström Laboratory, Uppsala University, P.O. Box 523, SE-75120 Uppsala, Sweden; gustav.ek@kemi.uu.se (G.E.); martin.sahlberg@kemi.uu.se (M.S.); 3 University Grenoble Alpes, CNRS, Institut Néel, 38000 Grenoble, France; laetitia.laversenne@neel.cnrs.fr (L.L.); nassif@ill.fr (V.N.)

**Keywords:** high-entropy alloys, hydrogen absorption/desorption, in situ neutron diffraction, cycling stability

## Abstract

Al_0.10_Ti_0.30_V_0.25_Zr_0.10_Nb_0.25_ was prepared to evaluate the effect of 10% aluminum into the previously reported quaternary alloy, Ti_0.325_V_0.275_Zr_0.125_Nb_0.275_. The as-cast quinary alloy formed a single-phase body centered cubic solid solution and transformed into a body centered tetragonal after hydrogenation. The alloy had a storage capacity of 1.6 H/M (2.6 wt.%) with fast absorption kinetics at room temperature, reaching full capacity within the first 10 min. The major improvements of Al addition (10%) were related to the desorption and cycling properties of the material. The temperature for hydrogen release was significantly decreased by around 100 °C, and the quinary alloy showed superior cycling stability and higher reversible storage capacity than its quaternary counterpart, 94% and 85% of their respective initial capacity, after 20 hydrogenation cycles without phase decomposition.

## 1. Introduction

Storing hydrogen in metals in the form of high capacity hydrides is an attractive alternative to high-pressure gas tanks, being a safe method with high energy density [1]. Yet, there are many challenges to overcome in metal hydrides for daily life applications due to, e.g., limited storage capacities (gravimetric and volumetric), harsh temperature and/or pressure conditions for hydrogen absorption/desorption reversibility, and limited life cycle (capacity fading). Different types of materials have been investigated for such purposes (Mg alloys, intermetallics, complex hydrides, etc.) but only recently high-entropy alloys (HEA) have acquired consideration for hydrogen storage applications. HEAs are a relatively new class of materials composed of five or more elements in near equimolar concentrations, 5–35 at.%, that can form single-phase solid solutions [2,3]. These materials often adopt simple crystalline structures such as body-centered cubic (*bcc*), face-centered cubic (*fcc*), or hexagonal close-packed (*hcp*), with the elements randomly distributed on a single crystallographic site. This is the reason why HEAs have a highly distorted lattice as compared to classical alloys [3]. This particular feature together with other core effects grant HEA remarkable mechanical properties such as high strength [4] and fracture resistance [5], superior to pure metals and some alloys, as well as other interesting physical properties like corrosion resistance [6] and superconductivity [7].

Recently, different groups have studied hydrogen sorption properties of these alloys and reported promising hydrogen storage capabilities. This is the case for TiVZrNbHf [8,9] that showed outstanding hydrogen capacity of 2.5 H/*M*, which is higher than its individual constituents binary hydrides or other classical refractory alloys. Most of the research in the literature are scattered attempts to find alloys with interesting hydrogenation properties [10,11,12,13,14,15,16], but only a few studies have tried to elucidate their properties in a more systematic approach as a function of the structural parameters, the chemical composition, or the electronic properties such as lattice distortion [17], valence electron concentration [18], and element composition [19,20,21]. In these previous works, the authors studied a series of high-entropy alloys by diverse structural, volumetric, and thermo-desorption techniques. The hydrogen sorption properties strongly depend on chemical composition. Being composed of at least five elements, HEAs possess many degrees of freedom in terms of chemical composition (number, nature and stoichiometry of elements within the HEA), and predicting their properties is far more complex than in classical alloys. Understanding these properties will allow the design of novel materials for hydrogen-related applications, a key factor for the design and development of new and efficient storing devices.

## 2. Results and Discussions

**Figure 1:**
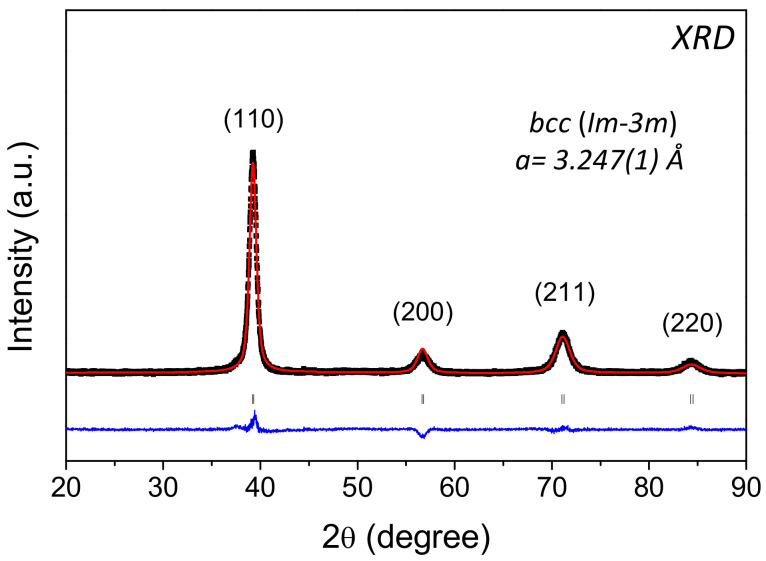
X-ray diffraction (XRD) pattern (λ = 1.54 Å) of the as-cast Al_0.10_Ti_0.30_V_0.25_Zr_0.10_Nb_0.25_ alloy and corresponding Rietveld refinement analysis.

The new HEA with composition Al_0.10_Ti_0.30_V_0.25_Zr_0.10_Nb_0.25_ was successfully synthesized by arc melting and adopts a *bcc* structure (*Im-3m*) with lattice parameter *a*_bcc_ = 3.247(1) Å and Rietveld analysis showing a good fitting for a single-phased solid solution (Figure 1). Similar results were reported for the quaternary alloy Ti_0.325_V_0.275_Zr_0.125_Nb_0.275_ (*a*_bcc_ = 3.261(1) Å) [22], see a comparison in Figure SI1 of the supporting information. The decrease of lattice constant by Al addition can be explained by the reduction of Zr concentration from 12.5 at.% for the quaternary to 10.0 at.% in the quinary composition, Zr being the largest element in the alloy (the elements Al, Ti, V, Zr, and Nb have the following atomic radii: 1.43, 1.45, 1.31, 1.59, and 1.43 Å, respectively [23]). Moreover, Al has comparable atomic size to Ti and Nb and consequently, the size disparity in the HEA is reduced and the lattice distortion, as defined previously in [24], decreases from *δ* = 6.0 to 5.5 for Ti_0.325_V_0.275_Zr_0.125_Nb_0.275_ and Al_0.10_Ti_0.30_V_0.25_Zr_0.10_Nb_0.25_, respectively.

**Figure 2:**
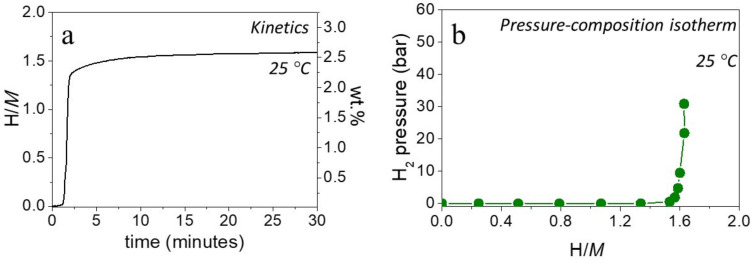
(**a**) Kinetics of absorption under 25 bar H_2_ pressure at 25 °C and (**b**) Pressure-composition isotherm at 25 °C of Ti_0.30_V_0.25_Zr_0.10_Nb_0.25_Al_0.10_.

The hydrogenation of Al_0.10_Ti_0.30_V_0.25_Zr_0.10_Nb_0.25_ was carried out in a single aliquot at room temperature, and the final equilibrium pressure was around 25 bar. The kinetic plot in Figure 2a shows rapid absorption after a short incubation time (~1 min) reaching almost full capacity within the first 5 min of hydrogen exposure. The maximum hydrogen capacity of the alloy was 1.6 H/*M* (2.6 wt.% gravimetric capacity). The PCI measurement in Figure 2b reveals a hydrogenation profile with a single step up to a maximum of 1.6 H/*M* and an equilibrium pressure in the range or below 10^−2^ bar at 25 °C. A precise value of the equilibrium pressure was not recorded due to the limits of detection of the pressure transducers (10^−2^ bar). A second pressure-compositions isotherms (PCI) measurement recorded at 100 °C also shows a single plateau with a low equilibrium pressure, see Figure SI2.

The same hydrogenation behavior was also noticed for the quaternary alloy but with a maximum hydrogen capacity of 1.8 H/*M* (2.5 wt.%) [22]. With the addition of 10 at.% of Al, the maximum hydrogen capacity of the material in terms of H/*M* decreased, but due to the lightweight property of Al, the quinary alloy had an increased gravimetric capacity (2.6 wt.%). Both alloys share a similar hydrogenation profile occurring in a single step with a low equilibrium pressure, thus, no significant effect in the destabilization of the phase by Al addition was noticed. Nevertheless, the HEA had fast absorption kinetics at room temperature after a mild activation treatment and possessed a comparable gravimetric capacity to other intermetallic compounds [25].

**Figure 3:**
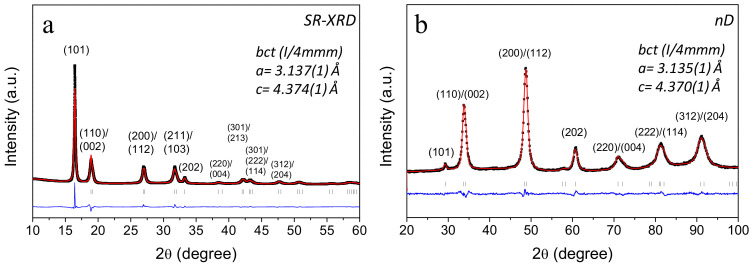
(**a**) Synchrotron radiation (SR)-XRD pattern (λ = 0.72896 Å) of the hydride Al_0.10_Ti_0.30_V_0.25_Zr_0.10_Nb_0.25_H_1.6_ and (**b**) ex situ neutron diffraction pattern (λ = 1.28 Å) of the deuteride Al_0.10_Ti_0.30_V_0.25_Zr_0.10_Nb_0.25_D_1.6_. The corresponding Rietveld refinements and *bct* lattice parameters are also given.

The structural properties of the hydride/deuteride phases were characterized by synchrotron radiation X-ray diffraction (SR-XRD) and neutron diffraction (nD), respectively (Table 1). The SR-XRD diffraction pattern of the hydride phase (1.6 H/*M*) in Figure 3a shows a single-phased hydride phase adopting a *bct* structure (*I4/mmm*) with the lattice parameters *a*_bct_ = 3.137(1) Å and *c*_bct_ = 4.374(1) Å, as determined from Rietveld analysis. The neutron diffraction pattern in Figure 3b also shows a good fitting with a *bct* structure with the lattice parameter *a*_bct_ = 3.135(1) Å and *c*_bct_ = 4.370(1) Å, in very good agreement with the SR-XRD results. The hydrogen/deuterium atoms were located in the tetrahedral interstitial sites of the *bct* structure ((0, 1/2, 1/4) and (0, 1/2, 3/4)), in agreement with previous studies [9].

**Figure 4:**
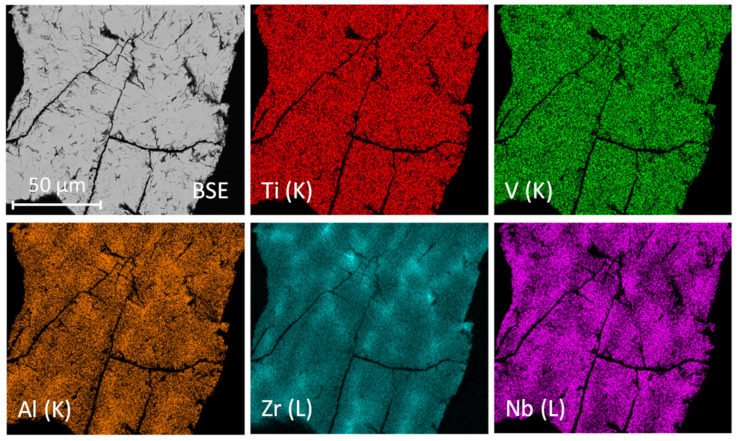
Chemical mapping, scanning electron microscopy and energy-dispersive X-ray spectroscopy (SEM-EDS) of the hydride Al_0.10_Ti_0.30_V_0.25_Zr_0.10_Nb_0.25_H_1.6_.

The chemical analysis by energy-dispersive X-ray spectroscopy (EDS) of the hydride phase in Figure 4 shows a homogeneous element distribution throughout the microstructure with the average alloy’s composition in good agreement with the nominal one (Table 2). However, some regions had slightly different atomic concentrations than the nominal composition (see Table 2). These concentration variations were minimal for Ti and V, but more visible for Al and Zr, which concentrate in the same regions (12.1 at.% Al, 17.5 at.% Zr), while slightly depleted in Nb (19.1 at.%). Both SR-XRD and nD analyses confirmed a single-phased hydride and therefore we suggest that these concentration variances were simple chemical modulations due to the solidification process. This peculiarity was not observed for the quaternary alloy. Thus, the addition of Al into the quaternary alloy triggered a small degree of local ordering of Al and Zr within the alloy’s microstructure.

**Figure 5:**
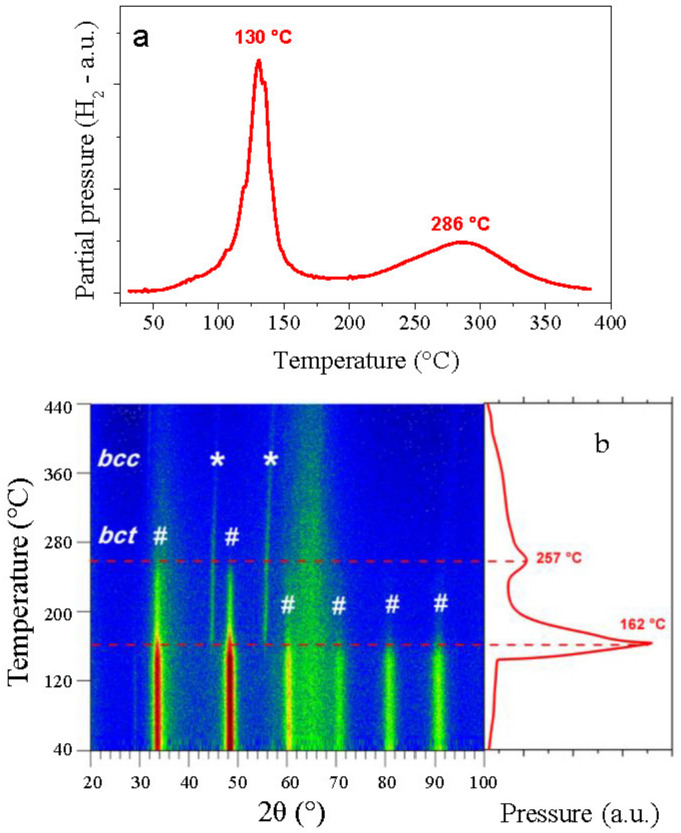
(**a**) Thermo-desorption spectroscopy (TDS) spectrum and (**b**) in situ neutron diffraction of Al_0.10_Ti_0.30_V_0.25_Zr_0.10_Nb_0.25_H(D)_1.6_ with a heating ramp of 1 °C/min under dynamic vacuum. The corresponding desorption profile during neutron diffraction is also shown on the right.

To further investigate the hydrogen desorption properties, thermo-desorption spectroscopy (TDS) analysis and in situ nD were performed during constant heating with 1 °C/min under dynamic secondary vacuum (Figure 5). The TDS profile in Figure 5a shows a main sharp desorption peak at around 130 °C with an onset temperature at 110 °C, followed by a second broad desorption event at 286 °C. For nD characterization, a deuterated sample was submitted to a constant heating rate of 1 °C/min while under dynamic vacuum, and the neutron diffraction data was collected every 15 s. Figure 5b contains the thermo-diffractogram obtained from 40 °C to 440 °C, along with the pressure variation in the secondary vacuum instrument.

The thermo-diffractogram shows the structural transformation of the HEA with increasing temperature from the hydride phase to a desorbed phase. At room temperature, the diffraction signals of the *bct* hydride, marked with hash symbols (#), were stable up to around 140 °C, where there was a sudden drop of the signal intensities. At this point, a new phase appeared with diffraction signals at around 45° and 55° in 2θ that corresponded to the *bcc* structure of the HEA, marked with the stars (*). Due to the low neutron cross-section of the elements involved, the intensity of the desorbed phase was low as compared to that of the initial deuteride [26]. This phase transformation is in good agreement with the pressure profile on the right, revealing a main desorption peak at around 160 °C. Both phases coexisted in the temperature range 160–250 °C while the intensity of the initial deuteride progressively decreased until a second minor desorption event occurred in the desorption profile at approximately 260 °C. Above this temperature, the diffraction signals of the hydride phase completely vanished, leaving only the desorbed *bcc* phase and the broad signals of the silica sample holder (32–36° and 60–70° in 2θ). The desorption profile showed a pressure tail after the second desorption peak at ~260 °C, and this was tightly related to the shrinking of the *bcc* lattice, as observed by the small shift of the diffraction peaks towards higher 2θ with increasing temperature. However, the nature of the second desorption peak remains unknown and might be tentatively related to the chemical modulation of the microstructure, as noticed in the EDS chemical mapping. It is worth noting that the TDS spectrum and the desorption profile during nD measurement are in good agreement, despite several tens of degree differences in the maximum of the peaks. However, this is not surprising, since different experimental systems have been used, as reported earlier [27].

Despite two desorption peaks in the desorption profile, these results verify that the HEA alloy underwent a single-phase transformation upon desorption and it can be inferred that the absorption process also occurred in a single-step transformation, in agreement with the PCI measurements above. Similar *bcc* HEAs (TiVZrNbHf [8,9], TiVZrNb [22], and TiZrHfMoNd [19]) have also been reported to have a single-phase transformation upon hydrogen absorption/desorption, unlike most classical *bcc* alloys which typically have two-phase transformations: metal ↔ monohydride ↔ dihydride [28,29].

As we propose to evaluate the effect of Al addition (10 at.%) in the composition Ti_0.325_V_0.275_Zr_0.125_Nb_0.275_, it is worth comparing the desorption behavior of the present Al_0.10_Ti_0.30_V_0.25_Zr_0.10_Nb_0.25_ and the quaternary alloy. The in situ nD measurement of the quaternary alloy recorded under similar experimental conditions is shown in Figure SI3. A phase transformation from a *fcc* dihydride to the *bcc* desorbed phase occurs abruptly at around 270 °C for the quaternary alloy, as described earlier [30]. Therefore, it is obvious that adding 10 at.% Al in the quaternary refractory alloy has a beneficial effect on the desorption temperature by drastically reducing the maximum desorption rate from 270 °C to 160 °C.

**Figure 6:**
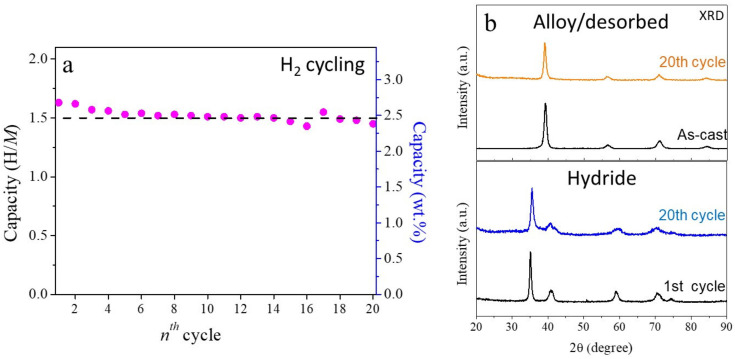
(**a**) Reversible hydrogen absorption capacity of Al_10_Ti_0.30_V_0.25_Zr_0.10_Nb_0.25_ upon 20 cycles at 25 °C and (**b**) the XRD patterns before and after 20 cycles as initial and desorbed phases (top) and hydride phases for the 1st and 20th cycles (bottom).

Lastly, the alloy was submitted to multiple hydrogen absorption/desorption cycles to evaluate the reversible storage capacity of the HEA. The cycling evaluation consisted in measuring the hydrogen capacity at room temperature, followed by complete hydrogen desorption. The latter step was done by heating to 400 °C while evacuating under secondary vacuum (10^−5^ mbar) for 10 h. The results are presented in Figure 6a for 20 hydrogenation cycles.

The Al_0.10_Ti_0.30_V_0.25_Zr_0.10_Nb_0.25_ had an initially capacity of 1.6 H/*M* (2.6 wt.%), which slightly decreased in the first five cycles. After that, the alloy seemed to reach a stable reversible capacity of ~1.5 H/*M* (2.45 wt.%). Moreover, the HEA alloy displayed a reversible gravimetric capacity of around 94% of its initial capacity. It is worth comparing these results (the reversible capacity in terms of gravimetric uptake and percentage of the initial capacity) to the quaternary composition prepared by arc melting and measured similarly. The latter alloy possessed a reversible hydrogen absorption capacity of 2.25 wt.% over 20 cycles, which represented approximately 85% of its initial capacity (2.7 wt.%), as depicted in Figure SI4. Obviously, the addition of Al into the quaternary composition increased the reversible gravimetric capacity of the material (from 2.25 to 2.45 wt.%) and improved the cycling stability (from 85 to 94% of the initial uptake). For the sake of comparison, Table 3 contains the capacities (initial and reversible, when reported) of several HEAs as weel as other intermetallics. The phenomenon of capacity fading by cycling is often encountered in metal hydrides, and detailed discussions can be found in our previous reports [30,31].

The alloy showed very good structural stability, maintaining the structures as well as the degree of crystallinity of the desorbed alloy and the hydride as single-phase *bcc* and *bct* lattices after 20 hydrogenation cycles, respectively, as demonstrated by the XRD patterns in Figure 6b. The lattice parameter of the desorbed phase after hydrogen cycling remained almost the same as the as-cast alloy. They hydride phase, on the other hand, had a small expansion of the *a*_bct_ parameter after 20 hydrogenation cycles (see Table 1). A SEM-EDS chemical analysis of the hydride phase after 20 hydrogenation cycles proved excellent chemical stability of the alloy by revealing similar chemical homogeneity as the initial state (Figure SI5).

In summary, the HEA with composition Al_0.10_Ti_0.30_V_0.25_Zr_0.10_Nb_0.25_ was successfully synthesized by arc melting in a single *bcc* phase with good chemical homogeneity. The alloy could rapidly absorb hydrogen, reaching full capacity of 1.6 H/M, or 2.6 wt.%, within the first 5 min of hydrogen exposure at room temperature. The crystalline structure underwent a reversible phase transformation upon hydrogen absorption/desorption from a pristine *bcc* to a hydride with *bct* lattice, as characterized by SR-XRD and nD. Due to the low equilibrium pressure for hydrogen absorption, the desorption of the hydride must be carried out at a high temperature under dynamic vacuum. The maximum desorption rate occurred at around 130 °C, which as significantly lower than 270 °C for its quaternary counterpart Ti_0.325_V_0.275_Zr_0.125_Nb_0.275_ or other related HEAs such as Mg_0.10_Ti_0.30_V_0.25_Zr_0.10_Nb_0.25_ (290 °C) [31] and Ti_0.30_V_0.25_Zr_0.10_Nb_0.25_Ta_0.10_ (180 °C) [30]. The HEA alloy possessed excellent hydrogen cycling properties with a reversible absorption capacity of 2.5 wt.%, which was significantly better than other similar high-entropy alloys [17,18] and conventional intermetalllics [25], see Table 3. Moreover, this alloy could withstand up to 20 hydrogenation cycles with a reversible storage capacity of 2.5 wt.% without phase decomposition, chemical segregation, or structure degradation.

## 3. Materials and Methods

The high-entropy alloy with composition Al_0.10_Ti_0.30_V_0.25_Zr_0.10_Nb_0.25_ was prepared by arc melting starting from coarse pieces of Ti (Alfa Aesar, 99.99%), Zr (Neyco, 99.95%), Nb (Alfa Aesar, 99.95%), V (Alfa Aesar, 99.70%), and Al (STREM Chem, 99%). All metals were mixed following the proposed stoichiometry and melted together in an arc furnace under Ar atmosphere and remelted 15 times, flipping the button over in between each melting to improve its homogeneity. The real (measured) chemical composition is listed in Table 2, showing minimal variation from the nominal composition.

Hydrogenation was carried out using Sievert’s method in an automatic PCTPro-2000 from SETARAM instrument or in a manual homemade manometric device with calibrated and thermostated volumes. About 500 mg of sample was sealed inside a stainless-steel sample holder, using metal gaskets to prevent gas leaking, and then connected to the vacuum rig of Sievert’s instrument. Before hydrogen exposure (grade N6), the sample was activated via a heat treatment at 340 °C for 2 h under dynamic vacuum (10^−5^ mbar). Pressure-compositions isotherms (PCI) and kinetics of absorption were measured at 25 °C.

The crystal structure of the pristine alloy and hydride phases were characterized by laboratory powder X-ray diffraction (XRD) and synchrotron radiation X-ray diffraction (SR-XRD), respectively. For laboratory XRD, the data was collected using a D8 Advance Bruker instrument with a Bragg–Brentano geometry, using Cu as the radiation source (λ(Cu K_α_) = 1.54 Å). The hydride phase was characterized by SR-XRD at the CRISTAL beamline (with capillary geometry) at SOLEIL synchrotron, France. For this analysis, the sample was finely grinded and loaded into a capillary of 0.2 mm diameter and probed with a radiation wavelength of *λ* = 0.72896 Å.

We have checked the repeatability of the synthesis, hydrogen sorption, and the laboratory XRD measurements. They were all repeatable, showing similar results.

Complementary structural characterization was carried out by in situ neutron diffraction technique (nD) on the hydride (deuteride) phase during deuterium desorption at the D1B beamline from the Institute Laue-Langevin, Grenoble, France https://doi.ill.fr/10.5291/ILL-DATA.CRG-2587, (accessed on 26 February 2020). In this experiment, a deuterated sample was loaded into a silica tube and connected to a vacuum rig with a base pressure of 10^−5^ mbar. The sample was then heated at a constant rate of 1 °C/min, from 25 to 650 °C, and the diffraction data was collected using a neutron wavelength of *λ* = 1.28 Å. For the precise localization of deuterium atoms into the hydride phase, ex situ nD powder diffraction was carried out at room temperature in a V sample holder.

The lattice parameters of the pristine and hydride phases were determined from XRD, SR-XRD, and ex situ nD diffraction patterns using the Rietveld method and Fullprof software [32]. The shape of the peaks was modeled using Thompson–Cox–Hastings pseudo-Voigt function.

The microstructure and chemical composition of the HEA was evaluated using scanning electron microscopy (SEM) and energy-dispersive X-ray spectroscopy (EDS). A representative portion of the hydride phase, without sieving or grinding, was immobilized in epoxy resin, polished, and subsequently coated with a Pd layer of 1.2 nm. The elemental analysis was carried out using an accelerated electron voltage of 10 keV with a beam current of 1 nA. The quantification of the elements was carried out using the signals from the electron shell of the elements Al(K), Ti(K), V(K), Zr(L), and Nb(L).

Hydrogen desorption properties were characterized by thermo-desorption spectroscopy (TDS) using a homemade instrument with a quadrupole mass spectrometer (QMS), Microvision Plus RGA, from MKS instruments, as described in earlier works [33]. About 10 mg of the hydride was loaded into the sample holder inside a high-purity Ar glovebox and then connected to a vacuum rig to minimize surface oxidation. The sample was heated at a constant rate of 1 °C/min while evacuating under secondary vacuum (10^−6^ mbar). The partial pressure of the desorbed H_2_ gas was recorded by QMS while constant heating.

Hydrogen storage performance was evaluated over 20 hydrogen absorption/desorption cycles to study reversible storage capacity. The absorption conditions consisted of exposing the alloy to a single dose of H_2_ for approximately 1 h or until equilibrium was reached, with a final pressure of ~25 bar at 25 °C. For hydrogen desorption, the hydride was heated to 400 °C and maintained constant for 10 h while under dynamic vacuum (10^−5^ mbar) to ensure full hydrogen desorption.

## 4. Conclusions

In conclusion, the addition of Al (10 at.%) into the quaternary composition Ti_0.325_V_0.275_Zr_0.125_Nb_0.275_ triggered a substantial improvement of the hydrogen sorption properties of the material, particularly by decreasing the temperature of hydrogen desorption and enhancing cycling stability. These insights demonstrated that the insertion of lightweight metals such as Al into refractory HEAs is beneficial for hydrogen storage performance. Owing to the vast compositional space of high-entropy alloys, this finding can help the design of novel HEAs with functional properties.

## Figures and Tables

**Table 1:** Lattice parameters of the Al_0.10_Ti_0.30_V_0.25_Zr_0.10_Nb_0.25_ alloy and corresponding hydride phase before and after 20 hydrogenation cycles. The lattice parameters of all phases were determined by the Rietveld refinement of diffraction patterns from laboratory XRD and SR-XRD for the hydride after the first hydrogenation.

Composition	Phase	Hydrogenation Cycle	Structure	Space Group	Lattice Parameters (Å)
Al_0.10_Ti_0.30_V_0.25_Zr_0.10_Nb_0.25_	as-cast	-	*bcc*	*Im-3m*	*a*_bcc_ = 3.247(1)
Al_0.10_Ti_0.30_V_0.25_Zr_0.10_Nb_0.25_H_1.6_	hydride	1st	*bct*	*I4/mmm*	*a*_bct_ = 3.137(1) *c*_bct_ = 4.374(1)
Al_0.10_Ti_0.30_V_0.25_Zr_0.10_Nb_0.25_	desorbed	20th	*bcc*	*Im-3m*	*a*_bcc_ = 3.244(1)
Al_0.10_Ti_0.30_V_0.25_Zr_0.10_Nb_0.25_H_1.5_	hydride	20th	*bct*	*I4/mmm*	*a*_bct_ = 3.197(1) *c*_bct_ = 4.368(1)

**Table 2:** Element concentrations of the hydride’s microstructure Al_0.10_Ti_0.30_V_0.25_Zr_0.10_Nb_0.25_H_1.6_, as determined by EDS.

Element	Nominal (at.%)	Measured (at.%)		
		Average	Al, Zr-Rich Region	Al, Zr-Poor Region
Al (K)	10	10.0 (0.6)	12.1 (0.6)	9.2 (0.4)
Ti (K)	30	29.5 (0.9)	27.8 (0.9)	29.8 (0.6)
V (K)	25	24.6 (1.2)	22.6 (1.2)	24.7 (0.7)
Zr (L)	10	10.8 (2.5)	17.5 (2.5)	8.3 (0.6)
Nb (L)	25	25.1 (1.6)	19.1 (1.6)	28.0 (0.5)

**Table 3:** Hydrogen storage capacities of several high-entropy alloys and intermetallics with their respective reversible capacity (if reported).

Alloy	Reference	Initial Storage Capacity		Reversible Storage Capacity	
		H/M	wt.%	H/M	wt.%
*Similar Ti-V-Zr-Nb HEAs*					
Ti_0.325_V_0.275_Zr_0.125_Nb_0.275_	[22]	1.8	2.7	1.5	2.25
Al_0.10_Ti_0.30_V_0.25_Zr_0.10_Nb_0.25_	present work	1.6	2.6	1.5	2.45
Mg_0.10_Ti_0.30_V_0.25_Zr_0.10_Nb_0.25_	[31]	1.7	2.7	1.5	2.4
Ta_0.10_Ti_0.30_V_0.25_Zr_0.10_Nb_0.25_	[30]	2.0	2.5	1.7	2.2
*Other equimolar HEAs*					
TiVZrNbHf	[8]	2.5	2.7	-	-
TiZrNbHfTa	[12]	2.0	1.7	-	-
TiVZrNb	[17]	2.0	2.7	-	-
TiVNb	[18]	2.0	3.0	0	0
TiVNbTa	[18]	1.9	2.0	0	0
TiVNbCr	[18]	2.0	3.1	1.2	2
*Intermetallics*					
TiFe	[25]	1.0	1.9	1.0	1.9
YFe3	[25]	1.2	1.9	1.2	1.9
LaNi5	[25]	1.1	1.5	1.1	1.5

## Data Availability

Not applicable.

## Supplementary Materials

Figure SI1. XRD pattern comparison between the quaternary Ti0.325V0.275Zr0.125Nb0.275 (top) and the quinary Al0.10Ti0.30V0.25Zr0.10Nb0.25 (bottom) alloys as-cast by arc melting technique. Figure SI2. Pressure composition isotherm of Al0.10Ti0.30V0.25Zr0.10Nb0.25H1.6 measured at 100 °C. Sample activated at 340 °C under dynamic vaccum for 2 hours. Figure SI3. In-situ neutron diffraction of Ti0.325V0.275Zr0.125Nb0.275D1.8 under a heating ramp of 1 °C/min while under dynamic vacuum, along with the corresponding desorption profile on the right. Figure SI4. Reversible hydrogen absorption capacity at 25 °C of Ti0.325V0.275Zr0.125Nb0.275 upon 20 cycles. The cycling evaluation consisted in measuring the hydrogen capacity at room temperature followed by complete hydrogen desorption. The latter step was done by heating at 400 °C while evacuating under secondary vacuum (10-5 mbar) for 10 hours. Figure SI5. Chemical mapping (SEM-EDS) of the hydride Al0.10Ti0.30V0.25Zr0.10Nb0.25H1.5 after 20 hydrogen absorption/desorption cycles. Similar Al-Zr-rich and Al-Zr-poor regions can be observed as the initial alloy.

## Sample Availability

Samples are available from the authors.
