# Supplementary material for: How 10 at% Al Addition in the Ti-V-Zr-Nb High-Entropy Alloy Changes Hydrogen Sorption Properties
Source: Molecules. 2021 Apr 23;26(9):2470. doi: 10.3390/molecules26092470 (PMC8122896; doi:10.3390/molecules26092470)

Supplementary Information

How 10 at% Al addition in the Ti-V-Zr-Nb high entropy alloy changes the hydrogen sorption properties?

Jorge Montero[1*], Gustav Ek[2], Laetitia Laversenne[3], Vivian Nassif[3], Martin Sahlberg[2], Claudia Zlotea[1*]

[1]*Univ Paris Est Créteil, CNRS, ICMPE, UMR 7182, 2 rue Henri Dunant, 94320 Thiais, France*

[2]*Department of Chemistry - Ångström Laboratory, Uppsala University, Box 523, SE-75120 Uppsala, Sweden*

[3]*University Grenoble Alpes, CNRS, Institut Néel, 38000 Grenoble, France*

* Corresponding authors: claudia.zlotea@icmpe.cnrs.fr and ing.monteroj@gmail.com

Content:

Figure SI1. XRD pattern comparison between the quaternary $Ti_{0.325}V_{0.275}Zr_{0.125}Nb_{0.275}$ (top) and the quinary $Al_{0.10}Ti_{0.30}V_{0.25}Zr_{0.10}Nb_{0.25}$ (bottom) alloys as-cast by arc melting technique.

Figure SI2. Pressure composition isotherm of $Al_{0.10}Ti_{0.30}V_{0.25}Zr_{0.10}Nb_{0.25}H_{1.6}$ measured at 100 °C. Sample activated at 340 °C under dynamic vaccum for 2 hours.

Figure SI3. *In-situ* neutron diffraction of $Ti_{0.325}V_{0.275}Zr_{0.125}Nb_{0.275}D_{1.8}$ under a heating ramp of 1 °C/min while under dynamic vacuum, along with the corresponding desorption profile on the right.

Figure SI4. Reversible hydrogen absorption capacity at 25 °C of $Ti_{0.325}V_{0.275}Zr_{0.125}Nb_{0.275}$ upon 20 cycles. The cycling evaluation consisted in measuring the hydrogen capacity at room temperature followed by complete hydrogen desorption. The latter step was done by heating at 400 °C while evacuating under secondary vacuum (10^{-5} mbar) for 10 hours.

Figure SI5. Chemical mapping (SEM-EDX) of the hydride $Al_{0.10}Ti_{0.30}V_{0.25}Zr_{0.10}Nb_{0.25}H_{1.5}$ after 20 hydrogen absorption/desorption cycles. Similar Al-Zr-rich and Al-Zr-poor regions can be observed as the initial alloy.

Figure SI1. XRD pattern comparison between the quaternary $Ti_{0.325}V_{0.275}Zr_{0.125}Nb_{0.275}$ (top) and the quinarlly $Al_{0.10}Ti_{0.30}V_{0.25}Zr_{0.10}Nb_{0.25}$ (bottom) alloys as-cast by arc melting technique.

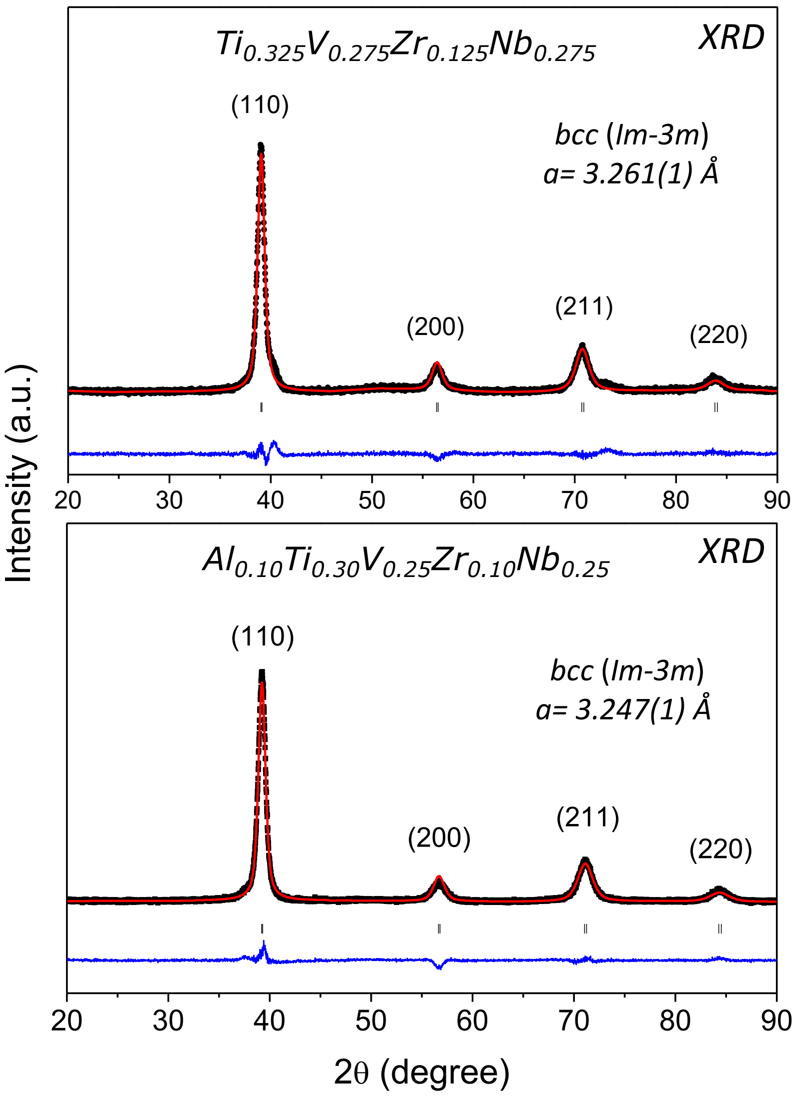

Figure SI2. Pressure composition isotherm of $Al_{0.10}Ti_{0.30}V_{0.25}Zr_{0.10}Nb_{0.25}H_{1.6}$ during measured at 100 °C. Sample activated at 340 °C under dynamic vaccum for 2 hours.

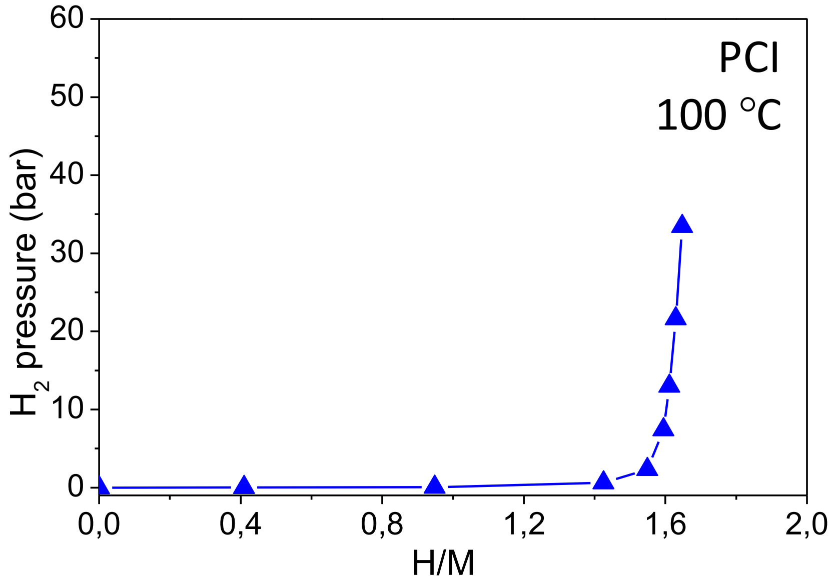

Figure SI3. *In-situ* neutron diffraction of $Ti_{0.325}V_{0.275}Zr_{0.125}Nb_{0.275}D_{1.8}$ under a heating ramp of 1 °C/min while under dynamic vacuum, along with the corresponding desorption profile on the right.

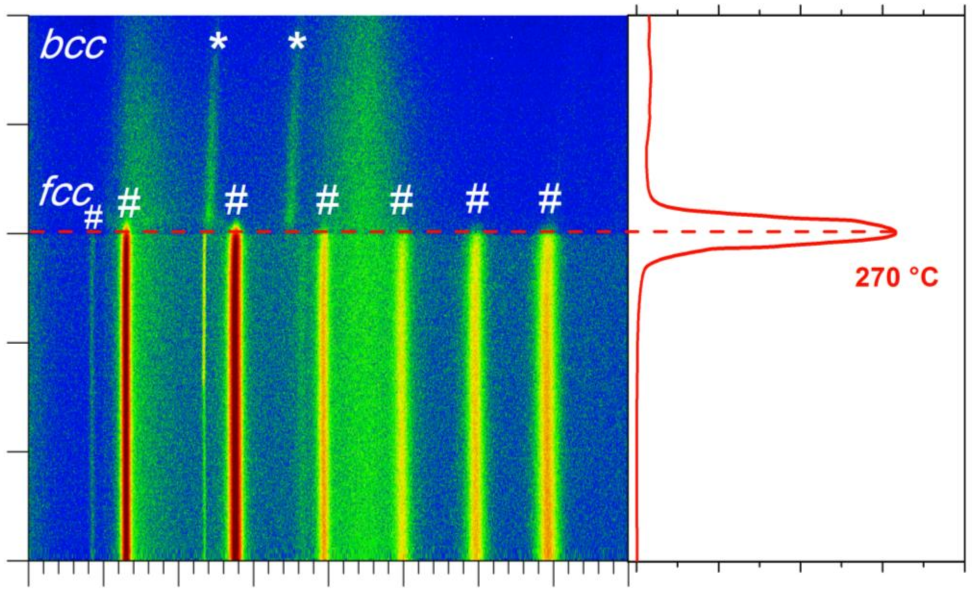

Figure SI4. Reversible hydrogen absorption capacity at 25 °C of $Ti_{0.325}V_{0.275}Zr_{0.125}Nb_{0.275}$ upon 20 cycles. The cycling evaluation consisted in measuring the hydrogen capacity at room temperature followed by complete hydrogen desorption. The latter step was done by heating at 400 °C while evacuating under secondary vacuum (10^{-5} mbar) for 10 hours.

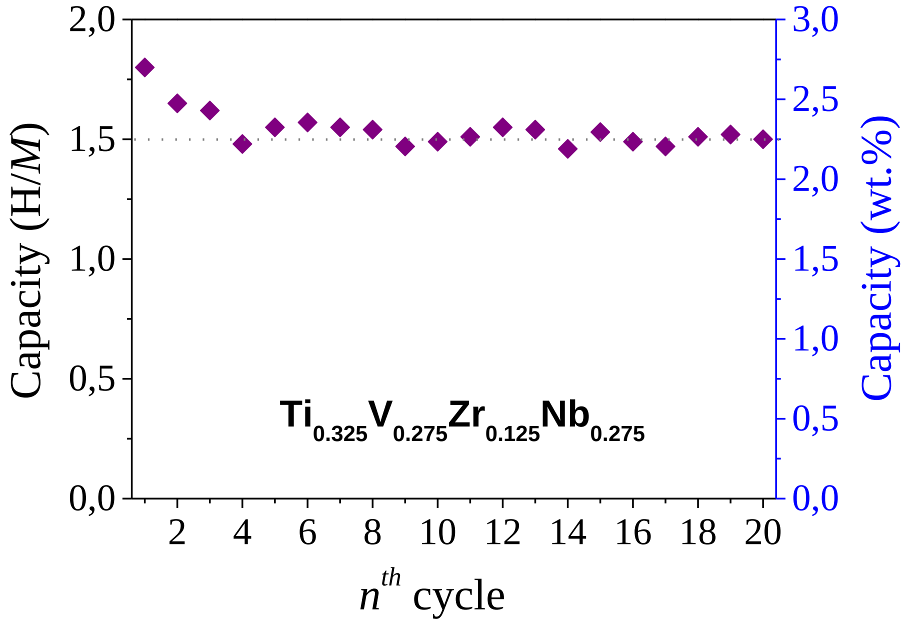

Figure SI5. Chemical mapping (SEM-EDX) of the hydride $Al_{0.10}Ti_{0.30}V_{0.25}Zr_{0.10}Nb_{0.25}H_{1.5}$ after 20 hydrogen absorption/desorption cycles. Similar Al-Zr-rich and Al-Zr-poor regions can be observed as the initial alloy.

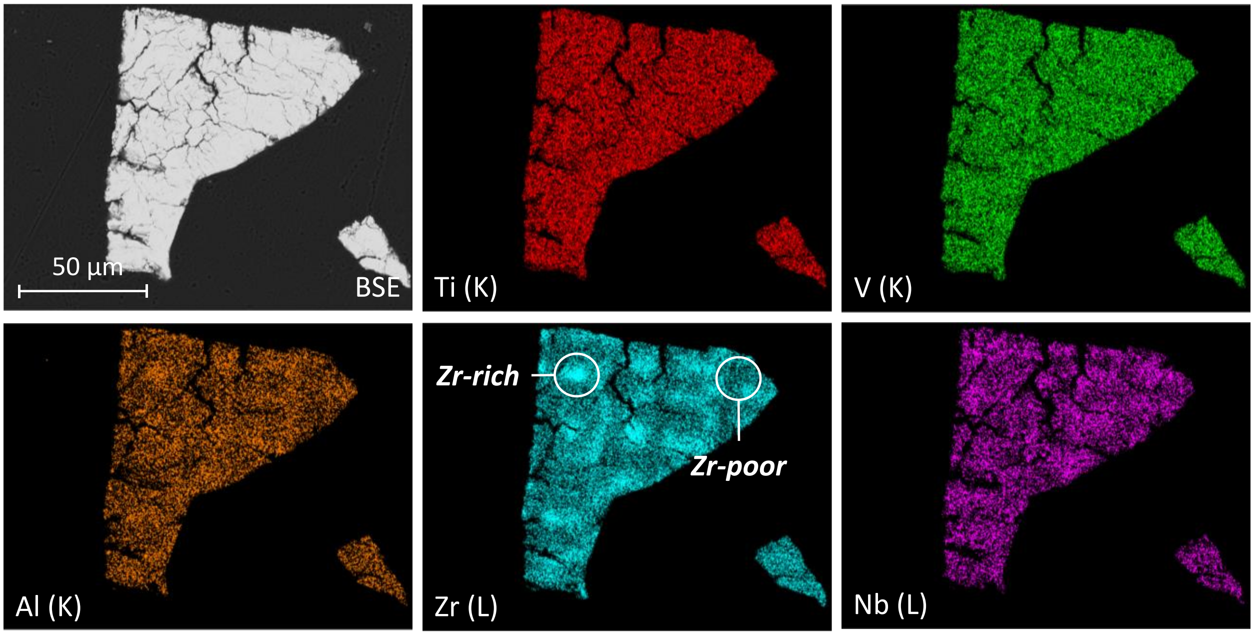

Supplement: Supplementary file 1 [file molecules-26-02470-s001.zip › molecules-1182562-supplementary.pdf]
